# Surface Charge-Modulated Toxicity of Cysteine-Stabilized Silver Nanoparticles

**DOI:** 10.3390/molecules29153629

**Published:** 2024-07-31

**Authors:** Magdalena Oćwieja, Anna Barbasz, Monika Wasilewska, Piotr Smoleń, Dorota Duraczyńska, Bogna D. Napruszewska, Mikołaj Kozak, Adam Węgrzynowicz

**Affiliations:** 1 Jerzy Haber Institute of Catalysis and Surface Chemistry, Polish Academy of Sciences, Niezapominajek 8, 30-239 Krakow, Poland; monika.wasilewska@ikifp.edu.pl (M.W.); piotr.smolen@ikifp.edu.pl (P.S.); dorota.duraczynska@ikifp.edu.pl (D.D.); bogna.napruszewska@ikifp.edu.pl (B.D.N.); 2 Department of Biochemistry and Biophysics, Institute of Biology and Earth Sciences, University of the National Education Commission, Podchorazych 2, 30-084 Krakow, Poland; anna.barbasz@up.krakow.pl; 3 Department of Physical Chemistry and Electrochemistry, Faculty of Chemistry, Jagiellonian University, Gronostajowa 2, 30-387 Krakow, Poland; mikolaj.kozak@doctoral.uj.edu.pl; 4 Institute of Organic Chemistry and Technology, Cracow University of Technology, Warszawska 24, 31-155 Krakow, Poland; adam.wegrzynowicz@pk.edu.pl

**Keywords:** silver nanoparticles, amino acids, cysteine, charge inversion, lymphocytes, cytotoxicity, streaming potential, zeta potential, genotoxicity, protein adsorption, impact of surface charge

## Abstract

The toxicity of silver nanoparticles (AgNPs) depends on their physicochemical properties. The ongoing research aims to develop effective methods for modifying AgNPs using molecules that enable control over the processes induced by nanoparticles in both normal and cancerous cells. Application of amino acid-stabilized nanoparticles appears promising, exhibiting tunable electrokinetic properties. Therefore, this study focused on determining the influence of the surface charge of cysteine (CYS)-stabilized AgNPs on their toxicity towards human normal B (COLO-720L) and T (HUT-78) lymphocyte cell lines. CYS-AgNPs were synthesized via the chemical reduction. Transmission electron microcopy (TEM) imaging revealed that they exhibited a quasi-spherical shape with an average size of 18 ± 3 nm. CYS-AgNPs remained stable under mild acidic (pH 4.0) and alkaline (7.4 and 9.0) conditions, with an isoelectric point observed at pH 5.1. Following a 24 h treatment of lymphocytes with CYS-AgNPs, concentration-dependent alterations in cell morphology were observed. Positively charged CYS-AgNPs notably decreased lymphocyte viability. Furthermore, they exhibited grater genotoxicity and more pronounced disruption of biological membranes compared to negatively charged CYZ-AgNPs. Despite both types of AgNPs interacting similarly with fetal bovine serum (FBS) and showing comparable profiles of silver ion release, the biological assays consistently revealed that the positively charged CYS-AgNPs exerted stronger effects at all investigated cellular levels. Although both types of CYS-AgNPs have the same chemical structure in their stabilizing layers, the pH-induced alterations in their surface charge significantly affect their biological activity.

## 1. Introduction

Silver nanoparticles (AgNPs) are well-known nanometric structures of silver widely used in diverse fields of science and industry [1,2,3]. The unusual biological activity of AgNPs has led to their numerous applications, especially in biology and medicine [4,5]. It has been established that AgNPs exhibit antibacterial, antifungal, and antiviral activity, making them suitable for the prevention and treatment of various diseases [6,7]. It is generally accepted that AgNPs are less biocidal towards eukaryotic cells than prokaryotic cells, creating a therapeutic window where mammalian cells remain unharmed while bacteria are effectively killed [8]. Based on this evidence, AgNPs have been considered a promising agent in the fight against the coronavirus SARS-CoV-2, which is responsible for the COVID-19 pandemic [9,10].

It should be noted that, in addition to their recent applications, AgNPs remain a focal point of interest for scientists searching for efficient anticancer agents [11,12]. Numerous literature reports have demonstrated the effectiveness of AgNPs in deactivating tumor cells and their utility in transporting anticancer drugs, addressing some of the challenges associated with conventional therapies. Furthermore, AgNPs can have a synergistic effect with anticancer drugs, allowing the use of lower doses. It is believed that AgNPs, serving as nanocarriers, provide less toxicity in healthy cells, potentially reducing the side effects caused by anticancer agents [13].

It seems plausible that the biological activity of AgNPs can be modeled by controlling their physicochemical properties. A series of scientific reports have shown that the biocidal and anticancer activities of AgNPs are strongly correlated with their size, morphology, surface charge, chemistry, and susceptibility to oxidative dissolution. Badawy et al. [14], in their investigation of the bactericidal properties of AgNPs on PolySeed (Gram-positive *bacillus* species), demonstrated that surface charge is one of the critical factors governing the toxicity of AgNPs. Abbaszadegan et al. [15] confirmed that positively charged AgNPs exhibit the highest level of effectiveness against selected Gram-positive and Gram-negative bacteria. Similar conclusions were reached by Suresh et al. [16], who investigated the impact of four types of AgNPs on lung epithelial cells and macrophage cells. Comparative cytotoxicity assessment revealed that positively charged poly(diallyldimethylammonium) chloride-stabilized AgNPs (PDDA-AgNPs) were more toxic than biogenic-AgNPs, oleate-AgNPs, and uncoated-AgNPs to both macrophage and lung epithelial cells [16]. Nevertheless, most studies on the toxicity of AgNPs have focused on negatively or positively charged AgNPs. Generally, various studies have shown that positively charged AgNPs, regardless of their size, are more toxic to both prokaryotic and eukaryotic cells [17,18,19,20,21,22]. However, research on the biological activity of AgNPs with tunable surface charges is rare and poorly documented in the literature. For instance, knowledge about the cytotoxicity of amino acid-stabilized AgNPs remains limited, despite the fact that the acid–base properties of amino acids, such as cysteine (CYS), can be transferred to AgNPs [23,24,25]. Studies have described the toxicity of cysteine-stabilized AgNPs towards various mammalian cells [23,24,25,26,27]. However, existing reports have not considered the effect of switching the surface charge of cysteine-stabilized AgNPs due to pH-dependent protonation and deprotonation of cysteine molecules on their biological activity. Moreover, the impact of this surface charge switch on the processes of protein corona formation has not been studied using the streaming potential method.

Considering the issues outlined above, the objective of this research was to synthesize cysteine-stabilized AgNPs (CYS-AgNPs), and determine their surface charge-dependent cytotoxicity towards a human normal B lymphocyte cell line (COLO-720L) and human T lymphocyte cell line (HUT-78). Selected cell types, such as lymphocytes, are crucial components of the human body’s defense system. These cells are widely distributed in blood and tissues and come into contact with “foreign” agents, including AgNPs. The body’s response to a toxic agent often depends on these cell types, which are among the first to encounter the foreign substance. For this purpose, the lymphocytes were exposed to positively or negatively charged CYS-AgNPs by controlling the pH of the applied suspensions. The response of the lymphocytes to the CYS-AgNP exposure was evaluated using numerous biochemical assays. The primary hypothesis was that the surface charge of cysteine-stabilized AgNPs would have a differential impact on various levels of cell functioning. It was expected that the crucial role of CYS-AgNP surface charge would predominantly manifest in their interactions with the cell membrane.

## 2. Results and Discussion

### 2.1. Characteristics of CYS-AgNPs Dispersed in Suspensions of Controlled pH

Cysteine-stabilized silver nanoparticles (CYS-AgNPs) were synthesized following a previously established protocol, involving the reduction of silver ions by sodium borohydride and subsequent modification of the newly formed nanoparticles’ negatively charged surface with L-cysteine (CYS) under acidic and anaerobic conditions [23]. Following synthesis, CYS-AgNPs were purified to remove unreacted compounds and dispersed in a solution with a pH of 4.0. The use of mild acidic conditions and storage of the purified suspension in a refrigerator at 4 °C helped prevent uncontrolled aggregation and destabilization of the CYS-AgNPs [23]. Silver content determination by ICP-OES allowed us to ascertain that the mass concentration of CYS-AgNPs in the stock suspension was equal to 120 ± 1 mg L^−1^. The recorded extinction spectra of CYS-AgNPs dispersed in suspensions with controlled pH values (regulated by nitric acid or Trizma buffer) revealed the appearance of a single absorption band. In the case of suspensions with a pH of 4.0, the maximum absorption band appeared at a wavelength of 401 nm, whereas at pH 7.4 and 9.0, it was localized at 397 nm (Supplementary Materials, Figure S1a).

In the next part of the study, CYS-AgNPs dispersed in suspensions with pH values of 4.0 and 9.0 were mixed with full RPMI1640 medium and the extinction spectra of these solutions were recorded again. Finally, these spectra were compared with the spectrum of RPMI1640 medium supplemented with 10% FBS. It is worth emphasizing that the final pH of each CYS-AgNP dispersion in full RPMI1640 medium was 7.4. This spectroscopic analysis revealed that the dispersion of CYS-AgNPs in the culture medium slightly changed the position of the characteristic absorption band (Figure S1b). In both cases, the introduction of CYS-AgNPs caused a bathochromic shift to a wavelength of 405 nm.

**Figure 1:**
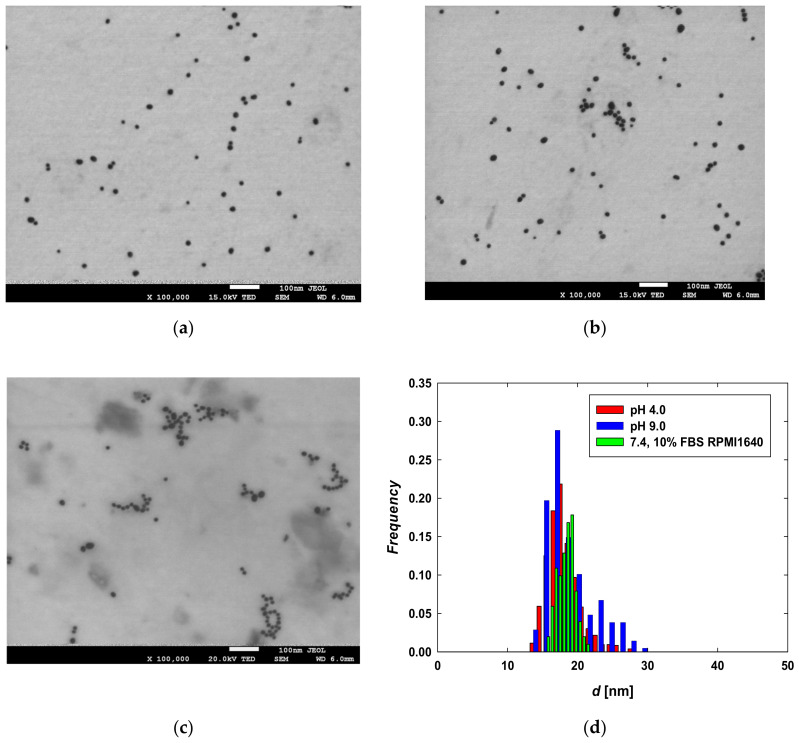
TEM micrographs of CYS-AgNPs dispersed in aqueous suspensions of (**a**) pH 4.0, (**b**) pH 9.0, and (**c**) in full RPMI medium supplemented by 10% FBS of pH 7.4; (**d**) size distribution of CYS-AgNPs after 24 h of incubation in pH 4.0 (red bars), pH 9.0 (blue bars) and 10% FBS RPMI 1640 medium (green bars). The histogram was constructed by analyzing the surface areas and diameters of 1000 CYS-AgNPs from the TEM micrographs.

The stock suspension was diluted to the mass concentration of CYS-AgNPs equal to 50 mg L^−1^ with the use of (a) nitric acid solution (pH 4.0), (b) Trizma buffer (pH 9.0), and (c) RPMI 1640 medium supplemented by 10% FBS (pH 7.4). Each solution containing CYS-AgNPs in a concentration of 50 mg L^−1^ was incubated at 37 °C over 24 h. Then, a drop (200 μL) of each suspension was placed on a copper grid covered by a thin carbon film. After the evaporation of the solvents, the samples were imaged with the use of an electron microscope. Typical TEM micrographs of CYS-AgNPs, following incubation in suspensions of varying pH and composition, are shown in Figure 1. As can be seen, regardless of the dispersive medium, CYS-AgNPs exhibit a quasi-spherical shape and a relatively narrow size distribution. It was determined that the average size of CYS-AgNPs at pH 4.0 and 9.0 was 18 ± 3 nm and 19 ± 4 nm, respectively. This finding was consistent with the average size of cysteine-stabilized AgNPs reported in previous studies [23,25], confirming the reproducibility of the synthesis method. Considering the calculated values of the polydispersity index (PdI) listed in Table 1, it was concluded that CYS-AgNPs were monodisperse. TEM micrographs of CYS-AgNPs dispersed in RPMI 1640 medium supplemented with 10% FBS also revealed quasi-spherical nanoparticles with an average size of 19 ± 2 nm. No aggregates of CYS-AgNPs were observed in the culture medium after 24 h of incubation. This observation confirms that CYS-AgNPs exist as individual nanoparticles in the culture medium and remain stable under the investigated conditions.

CYS-AgNPs dispersed in aqueous solutions were characterized using dynamic light scattering (DLS) and electrophoretic light scattering (ELS) techniques at a temperature of 37 °C, which is typical for biological tests involving diverse culture cells. The dependencies of the CYS-AgNP diffusion coefficient and hydrodynamic diameter on ionic strength, regulated by the addition of sodium chloride and sodium nitrate, are presented in the Supplementary Materials (Figure S2). The measurements were conducted at pH 4.0, 7.4, and 9.0, regulated by Trizma buffer. It was observed that the hydrodynamic diameter of CYS-AgNPs remained unchanged for ionic strengths below 8·10^−3^ M at pH 4.0 and 9.0. Additionally, it was found that at 37 °C, CYS-AgNPs were more sensitive to the presence of sodium chloride than sodium nitrate. These results were consistent with data described for cysteine-stabilized AgNPs dispersed in aqueous suspensions at a temperature of 25 °C [24]. CYS-AgNPs were the least stable at pH 7.4, where an increase in the hydrodynamic diameter of CYS-AgNPs, indicating an aggregation process, was observed at an ionic strength of 4·10^−3^ M.

It was observed that CYS-AgNPs were unstable in pure RPMI 1640 medium, which is characterized by high electrical conductivity and an ionic strength higher than 0.1 M. Determining the hydrodynamic diameter of CYS-AgNPs in RPMI 1640 medium supplemented with 10% FBS using the DLS technique was not feasible due to the approximately 2000-fold higher concentration of protein molecules relative to the AgNP concentration. However, based on TEM micrographs of CYS-AgNPs dispersed in the supplemented medium, it can be inferred that the excess of protein molecules relative to AgNPs prevents their uncontrolled aggregation. The selected physicochemical properties of AgNPs dispersed in the aqueous media are summarized in Table 1.

The experimental conditions for the electrokinetic studies were identical to those used for measuring the diffusion coefficients of CYS-AgNPs (Figure S3). It was observed that at pH 4.0, the electrophoretic mobility of CYS-AgNPs decreased significantly with an increase in ionic strength (Figure S3a). The values of the zeta potential of CYS-AgNPs, calculated based on Henry’s model [25], decreased from 69 ± 2 mV for an ionic strength of 10^−4^ M NaCl to 28 ± 3 mV for an ionic strength of 10^−2^ M (Table 1).

At pH 7.4 and 9.0, CYS-AgNPs exhibited negative charges. At these pH values, the zeta potential of CYS-AgNPs increased with the rise in ionic strength (Figure S3b). Conversely, for a fixed ionic strength, the zeta potential values were more negative at higher pH levels (Figure S3b). Considering the aggregation of CYS-AgNPs in RPMI 1640 medium and the protein molecule excess relative to CYS-AgNPs in full RPMI 1640 medium, the electrokinetic properties were not determined in this colloidal system.

It was confirmed that the electrokinetic properties of CYS-AgNPs (Table 1) are consistent with previously reported findings [24]. Furthermore, it was determined that the isoelectric point of CYS-AgNPs at a temperature of 37 °C also occurs at pH 5.1 [25]. For clarity, CYS-AgNPs characterized by positive and negative values of zeta potential were labeled as CYS-AgNPs(+) and CYS-AgNPs(−), respectively. It is worth mentioning that the properties of CYS-AgNPs at the isoelectric point were not determined. Previous works [23,24,25] have shown that at pH 5.1, CYS-AgNPs aggregate. It is well known that using unstable colloidal suspensions is impractical because they lead to non-reproducible results in biological tests. Therefore, for further studies, CYS-AgNPs dispersed in suspensions at pH 4.0 and 9.0 were selected. Under these conditions, they were stable and exhibited diverse surface charges despite having chemically identical stabilizing layers.

The stability of CYS-AgNPs was also studied in relation to their oxidative dissolution and release of silver ions. For this purpose, two suspensions of CYS-AgNPs, with a concentration of 50 mg L^−1^ and pH values of 4.0 and 9.0, were maintained at a temperature of 37 °C for 48 h. The concentration of leached silver ions was determined at specific time intervals, which enabled the determination of kinetics of silver ion release. The results of these studies, presented in the Supplementary Materials (Figure S4), showed that initially the dissolution of CYS-AgNPs at pH 4.0 was faster than at pH 9.0. Nevertheless, after 15 h, in both cases, the concentration of leached silver ions stabilized at a constant value of 8.5 mg L^−1^ (Figure S4).

### 2.2. Determination of Interactions between CYS-AgNPs and Protein Molecules

The next aim of the research was to determine the impact of CYS-AgNP charge on the adsorption process of various protein molecules on the nanoparticle surface. These studies were oriented to investigate the adsorption process of biotin-labeled BSA (Bt-BSA) and molecules from FBS on the surfaces of positively and negatively charged CYS-AgNPs. In other words, the aim was to determine whether protonation or deprotonation of cysteine molecules on the CYS-AgNP surface occurs, or if cysteine molecules participate in the formation of a protein corona around the silver core. To achieve this, UV-vis spectroscopy and streaming potential measurements were employed.

**Figure 2:**
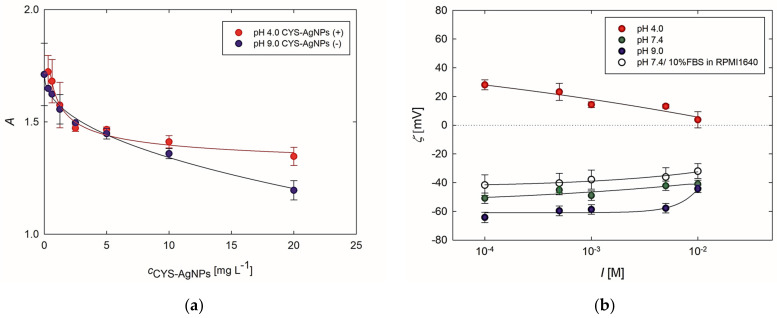
(**a**) Effect of Bt-BSA deposition on CYS-AgNPs determined as the dependence of absorption (at λ = 450 nm) of Bt-BSA on CYS-AgNPs(+) or CYS-AgNP(−). (**b**) Effect of FBS deposition on CYS-AgNP monolayers determined as the dependence of zeta potential of monolayers on ionic strength at selected pH based on the streaming potential measurements. The solid lines are a fit of the experimental data.

A well-known assay based on measuring changes in the absorbance of Bt-BSA at 450 nm wavelength, due to interactions with AgNPs, was used to evaluate potential interactions between CYS-AgNPs and BSA as a model protein [20]. The results of these studies are presented in Figure 2a. Analyzing these data reveals that the absorbance decreases with an increase in CYS-AgNP concentration. Specifically, up to the CYS-AgNPs concentration of 10 mg L^−1^, the changes in the values of absorbance are comparable for both cases. In turn, at a concentration of 20 mg L^−1^, CYS-AgNPs(+) induce a ca. 12% lower decrease in absorbance than CYS-AgNPs(−). Therefore, a higher absorbance indicates that more Bt-BSA molecules bound to CYS-AgNPs(+). Nevertheless, these findings provide evidence that attractive interactions also occur between Bt-BSA and CYS-AgNPs(−). It is worth mentioning that the adsorption of Bt-BSA on negatively charged AgNPs obtained with the use of tannic acid was also reported in the literature [20]. Moreover, it was previously established that the binding interactions between negatively charged citrate-stabilized AgNPs and BSA are mainly driven by hydrophobic forces [28,29]. Combining the aforementioned evidence with the data, one can conclude that Bt-BSA deposits on both forms of CYS-AgNPs.

Additionally, the interactions of CYS-AgNPs with FBS present in culture medium were assessed using the electrokinetic measurements. As previously stated, the determination of FBS and CYS-AgNP interactions in the full culture medium with the use of electrophoretic mobility measurements proved impractical due to a substantial excess of FBS molecules approximately 2000-fold greater than the CYS-AgNPs. Hence, it can be inferred that the determined electrophoretic mobility should be attributed to the protein molecules rather than to CYS-AgNPs. To circumvent this issue, positively and negatively charged CYS-AgNPs were immobilized on oppositely charged planar surfaces, resulting in monolayers with the highest possible coverage achievable at an ionic strength of 5 × 10^−3^ M NaCl. Based on the SEM micrographs of the recorded CYS-AgNP monolayers (Supplementary Materials, Figure S5), it was determined that the dimensionless coverage was equal to 25% and 23% for the CYS-AgNP(+) and CYS-AgNP(−) monolayers, respectively. As depicted in Figure 2, the CYS-AgNP monolayers investigated at pH 4.0 exhibited a positive charge, whereas at pH 7.4 and 9.0, they were negatively charged. For pH 7.4 and 9.0, the zeta potential of CYS-AgNP monolayers notably increased with ionic strength, consistent with previously described data [25].

It is noteworthy that at pH 7.4, the zeta potential of CYS-AgNPs deposited as monolayers exhibited lower negative values than at pH 9.0 across the entire range of ionic strength. Furthermore, it increased from −51 ± 4 mV at an ionic strength of 10^−4^ M to −41 ± 2 mV at an ionic strength of 10^−2^ M. Conversely, the 24 h exposure of the CYS-AgNP monolayers to contact with RPMI 1640 culture medium supplemented by 10% FBS led to a further increase in the value of the zeta potential across the entire range of ionic strengths (Figure 2). SEM micrographs revealed that the contact of CYS-AgNP monolayers with full culture medium does not induce desorption of nanoparticles from the formed monolayers (Figure S5c,d). Therefore, one can deduce that the observed dependence results from the deposition of FBS molecules on the CYS-AgNP monolayers. These findings are also consistent with the spectrometric investigations conducted using Bt-BSA (Figure 2a). Both types of studies confirmed that protein molecules interact with positively and negatively charged CYS-AgNPs. UV-vis measurements revealed a higher quantity of deposited proteins on positively charged CYS-AgNPs (Figure 2a). Moreover, the streaming potential measurements showed that introducing CYS-AgNPs(+) with a positive zeta potential, as well as CYS-AgNPs(−) with a negative zeta potential, into the full culture medium causes the deposition of FBS molecules on their surfaces. Consequently, it was established that both forms of CYS-AgNPs dispersed in the full culture medium exhibit a negative zeta potential due to the deposition of negatively charged FBS molecules on their surface. Additionally, it was demonstrated that the acid–base properties of cysteine molecules adsorbed on the CYS-AgNP surface influence the process of protein corona formation.

### 2.3. Biological Activity of CYS-AgNPs Towards Lymphocytes

The evaluation of CYS-AgNP biological activity towards COLO-720L and HUT-78 cells was conducted considering that these AgNPs can be positively or negatively charged depending on the pH values. To further address this issue, CYS-AgNPs were introduced into the full culture medium using suspensions at pH 4.0 and 9.0, where they exhibit positive and negative charges, respectively (Figure S3) [24]. After 24 h exposure of the lymphocytes on CYS-AgNPs, several biochemical assays and microscopic imaging were applied to determine the biological and morphological changes in the treated cells.

**Figure 3:**
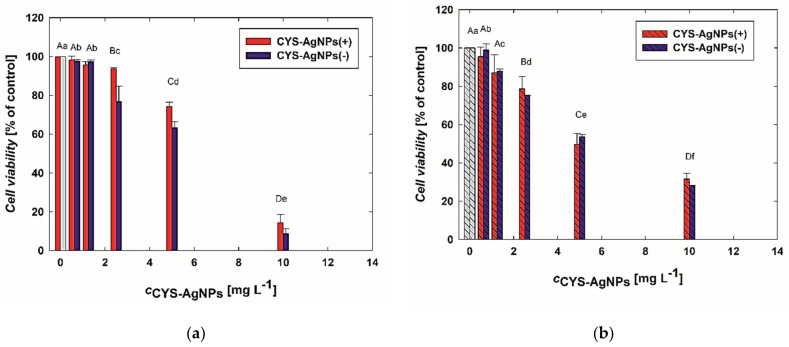
The impact of CYS-AgNPs on the viability of (**a**) COLO-720L and (**b**) HUT-78 cells was assessed. The viability of the lymphocytes was evaluated after 24 h of CYS-AgNP treatment using the MTT assay. Data obtained for control samples (without CYS-AgNPs) are marked in gray. Data points are means ± SD (five replicate trials). Different letters indicate significant (*p* < 0.05) differences between treatments.

In the first step, concentration-dependent changes in the viability of the cells were investigated based on the results gathered from the MTT assay. The results of this part of the study are presented in Figure 3. It is noticeable that the viability of lymphocytes significantly decreases with an increasing concentration of CYS-AgNPs. Interestingly, CYS-AgNPs(−) at higher concentrations (>5 mg L^−1^) induced a noticeably higher decrease in the viability of COLO-720L cells than CYS-AgNPs(+). For instance, after exposure to CYS-AgNPs(−) and CYS-AgNPs(+) at a concentration of 10 mg L^−1^, the COLO-720L viability decreased to 8% and 14%, respectively (Figure 3a). Moreover, it can be observed that COLO-720L cells were more sensitive to the CYS-AgNP treatment than HUT-78 cells.

Analyzing Figure 3b, it is evident that HUT-78 cells appear to be more susceptible to the action of CYS-AgNPs. The viability of HUT-78 cells is also dependent on the CYS-AgNP concentration. Treatment with a dose of 10 mg L^−1^ resulted in a viability drop to 31% and 28% for CYS-AgNPs(−) and CYS-AgNPs(+), respectively. Furthermore, the impact of CYS-AgNP surface charge on the mitochondrial activity of HUT-78 cells appears to be insignificant.

**Figure 4:**
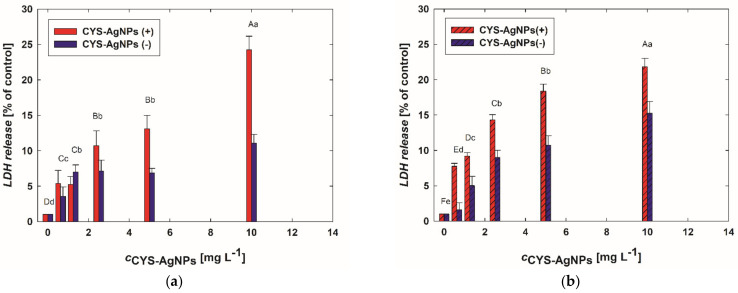
Impact of CYS-AgNPs on the LDH release and membrane integrity of (**a**) COLO-720L and (**b**) HUT-78 cells. The LDH release from the lymphocyte cells was evaluated after 24 h of CYS-AgNP treatment using LDH assay. Data points are means± SD (five replicate trials). Different letters indicate significant (*p* < 0.05) differences between treatments.

The disruption of the cell membrane after CYS-AgNP treatment was assessed by measuring the secretion of LDH. The data presented in Figure 4 show that CYS-AgNPs strongly affect the integrity of cell membranes. Significant release of LDH as a result of exposure to CYS-AgNPs was detected even at a concentration of 0.6 mg L^−1^. Similarly, as observed in the assessment of concentration-dependent changes in cell viability detected based on mitochondrial activity (Figure 3), LDH release increases with CYS-AgNP concentration. However, the impact of CYS-AgNP surface charge on cell membrane destruction is significant. It was observed that CYS-AgNPs(+) induce LDH secretion more strongly than CYS-AgNPs(−). This dependence is visible for both types of lymphocytes.

It is worth mentioning that previous results (Figure 2) showed that positively and negatively charged CYS-AgNPs are covered by proteins from FBS-supplemented medium. It was found that the surface charge of CYS-AgNPs introduced to the culture medium influences their interactions with proteins and consequently affects the observed toxicity. Both basic cell viability tests (MTT and LDH) showed a clear difference in lymphocyte sensitization to CYS-AgNPs. COLO-720L exhibited a greater decrease in viability compared to HUT-78. The differences in the lymphocyte response to the CYS-AgNP treatment were especially visible in the results of LDH tests. However, these findings are not surprising because the previous literature demonstrates that susceptibility to cell membrane damage due to nanoparticle treatment is closely correlated with the lipid composition of the membrane [30].

A highly destructive action of CYS-AgNP(+) was also observed in the DNA damage (Supplementary Materials, Figure S6). A nearly six-fold higher DNA disruption compared to the control was detected in COLO-720 cell after 24 h of exposure to CYS-AgNPs(+). In turn, CYS-AgNPs(−) caused two-fold less damage than CYS-AgNPs(+) when applied at the same concentration of 10 mg L^−1^ (Figure S6).

### 2.4. The Changes in the Lymphocyte Morphology as a Result of the CYS-AgNP Treatment

To assess the impact of CYS-AgNPs on the morphology of both types of lymphocytes, the cells were treated with nanoparticles at concentrations of 5 and 10 mg L^−1^. After 24 h of exposure to the CYS-AgNPs, cell samples were prepared and imaged using TEM. For comparison, samples of untreated lymphocytes were also prepared for TEM imaging.

**Figure 5:**
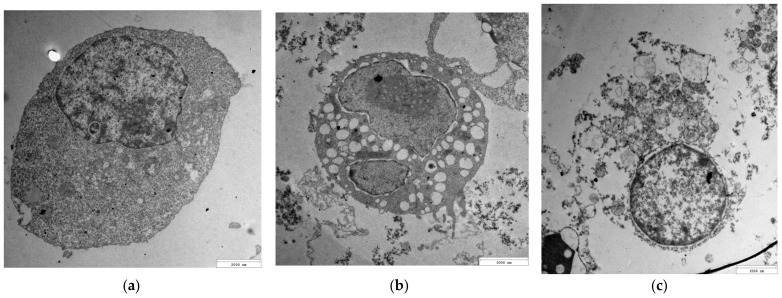
TEM micrographs of COLO-720L cells: (**a**) before and after 24 h of exposure to (**b**) CYS-AgNPs(+) and (**c**) CYS-AgNPs(−) at concentrations of 5 mg L^−1^. The scale bar represents 3000 nm.

**Figure 6:**
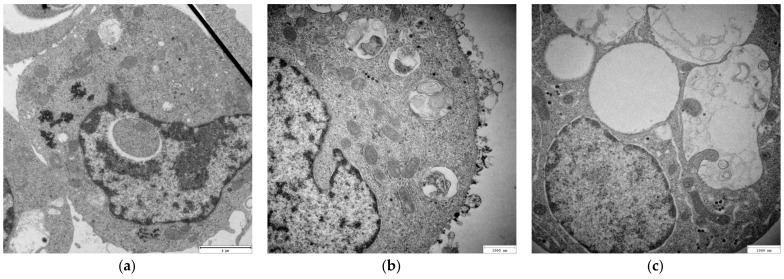
TEM micrographs of HUT-78 cells: (**a**) before and after 24 h of exposure to (**b**) CYS-AgNPs(+) and (**c**) CYS-AgNPs(−) at a concentration of 10 mg L^−1^. The scale bar represents 2000 nm for panel (**a**) and 1000 nm for panels (**b**) and (**c**).

As shown in Figure 5 and Figure 6, the control samples of the cells did not reveal any abnormalities. In the case of CYS-AgNP-treated lymphocytes, the endosomes near the cell membrane with a large number of big and black aggregates were detected. Characteristic aggregates of CYS-AgNP were found to distribute throughout the cytoplasm, inside lysosomes and the nucleus. Specific inclusions of CYS-AgNPs found inside endosomes and in cytoplasm were similar to those of aggregates. However, magnified images showed the presence of individual CYS-AgNPs. Large endosomes with characteristic inclusions were observed in the cytoplasm of the lymphocytes, as well as near the cell and nuclear membrane, suggesting that CYS-AgNPs were entering the cells through endocytosis rather than diffusion. The cytoplasm of the cells exhibited multiple endosomes containing engulfed nanoparticles, with similar endosomes also observed near the nuclear membrane. This finding is consistent with results described by AshaRani et al. [31]. Comparing the micrographs recorded for both types of cells, it is evident that exposure to CYS-AgNPs(+) caused more significant changes in COLO-720L cells than in HUT-78 cells. Regardless of the visualization location, COLO-720L cells exhibited more varied-sized holes and discontinuities in the cell membranes. The application of negatively-charged CYS-AgNPs also resulted in total cell damage. In this case, COLO-720L cells were more sensitive to the application of CYS-AgNPs(−) than HUT-78 cells. Significant morphological changes in COLO-720L cells were detected at lower CYS-AgNP concentrations than in HUT-78 cells. Overall, the morphological changes induced by both types of CYS-AgNPs were strongly dose-dependent. Estimating the charge-dependent uptake of CYS-AgNPs based on the recorded TEM micrographs was not possible. It is worth noting that the quantitate evaluation of the intracellular content of CYS-AgNPs was also not achievable. The results obtained from ICP-OES were not reproducible.

In conclusion, it should be mentioned that various studies have revealed that positively charged AgNPs, regardless of their size, are more toxic to both prokaryotic and eukaryotic cells. Silva et al. [17], investigating the toxicity of three types of organo-coated AgNPs towards *Escherichia coli* and *Daphnia magna*, discovered that positively charged AgNPs covered by branched polyethyleneimine (bPEI) were the most harmful to the tested organisms. Similarly, Kasements et al. [18], studying the impact of AgNPs on *Saccharomyces cerevisiae* BY4741, determined that bPEI-AgNPs characterized by a positive surface charge were 8–44 times more toxic than negatively charged citrate-stabilized AgNPs. Zhang et al. [19] established that the surface charge of AgNPs mediates the accumulation dynamics in *Chlorella vulgaris*. The authors found that the uptake rate constant of positively charged PEI-AgNPs was approximately 20 times higher than that determined for negatively charged AgNPs stabilized by citrate anions. Zhang et al. [19] observed that the positively charged AgNPs were more effectively bioaccumulated than their negatively charged counterparts.

However, these correlations between AgNP surface charge and their toxicity are not universally applicable. Barbasz et al. [20] conducted studies on the toxicity of three types of AgNPs characterized by similar morphology and silver ion release profile but diverse surface properties towards histiocytic lymphoma (U-937) and human promyelocytic cells (HL-60). The obtained outcomes did not confirm that positively charged AgNPs stabilized by cysteamine are the most toxic for the investigated cells. Based on the analysis of results gathered from several biochemical assays, it was concluded that negatively charged AgNPs obtained with the use of sodium borohydride were the most destructive for the tumor cells [20].

Interesting and valuable studies were also carried out by Kubo et al. [21], who assessed the antibacterial potency of differently coated 10 and 50 nm AgNPs against the clinically relevant bacteria *Escherichia coli* and *Staphylococcus aureus*. The investigation included negatively and positively charged AgNPs coated by citrate anions, bis-2-ethylhexyl sulfosuccinate (AOT), polyvinylpyrrolidone (PVP), polysorbate 80 (Tween 80), cetyltrimethylammonium bromide (CTAB), and poly-L-lysine (PLL). The results obtained by Kubo et al. [21] unequivocally showed that, contrary to the common belief, positively charged AgNPs did not exhibit superior antibacterial properties compared to the negatively charged AgNPs. The authors suggested that solubility might be a more important determinant of antibacterial activity than the surface charge itself. Similar conclusions regarding the impact of AgNP surface charge on their biocidal properties were drawn by Gibała et al. [21], who investigated the biological activity of AgNPs stabilized by low molar mass compounds other than those described by Kubo et al. [21]. The research of Gibała et al. [22] involved four types of positively charged AgNPs stabilized by lysine (LYZ), arginine (ARG), cysteine (CYS), and cysteamine, and eleven types of negatively charged AgNPs obtained with the use of diverse organic and inorganic compounds. It was established that arginine-stabilized AgNPs were the most biocidal among those investigated; however, some other positively charged AgNPs were found to be less toxic than certain negatively charged AgNPs. Nevertheless, in the studies presented by Gibała et al. [22], the impact of the charge-switchable properties of amino acid-stabilized AgNPs on their biocidal activity was not considered. This report demonstrated that cysteine molecules chemisorbed on the surface of AgNPs [23] influence the surface charge of AgNPs due to the protonation and deprotonation of amine and carboxyl moieties. When CYS-AgNPs are introduced into the culture medium in either a positively or negatively charged form, they induce different effects in the two lymphocyte lines tested. Therefore, the results show that despite having the same chemical structure in their stabilizing layer, AgNPs can exhibit diverse biological activities towards the tested cells due to the influence of external factors such as pH, which alters their surface charge.

## 3. Materials and Methods

### 3.1. Chemicals

Silver nitrate, sodium borohydride, L-cysteine, 2-amino-2-(hydroxymethyl)-1,3-propanediol (Trizma^®^ base), anhydrous ethanol, and nitric acid were obtained from Sigma-Aldrich, Saint Louis, MO, USA. Poly(diallyldimethylammonium chloride), hereafter referred to as PDDA, with a weight-averaged molar mass of Mn = 160,000 g mol^−1^ and number-averaged molar mass of Mw = 101,000 g mol^−1^, was purchased from Polymer Standard Service GmbH, Mainz, Germany. Quantities of 2.5% glutaraldehyde in 0.1-M cacodylate buffer and osmium tetroxide ethanol Poly/Bed 812 were obtained from Polysciences, Warrington, PA, USA. The chemicals were of analytical grade and used without further purification. Ultrapure water (Milli-Q water) with a conductivity of 0.06 μS cm^−1^ was obtained using a Milli-Q Elix & Simplicity 185 purification system (Millipore SA, Molsheim, France). Natural ruby mica sheets used in the deposition experiments of CYS-AgNPs were supplied by Continental Trade^®^, Warsaw, Poland.

The human normal B lymphocyte cell line (COLO-720L) and human T lymphocyte cell line (HUT-78) were purchased from the European Collection of Cell Cultures via Sigma Aldrich, Saint Louis, MO, USA. RPMI 1640 culture medium (with L-glutamine), fetal bovine serum (sterile-filtered, suitable for cell culture), and antibiotic solution (10,000 U mL^−1^ penicillin, 10 mg mL^−1^ streptomycin) were obtained from CytoGen GmbH, Greven, Germany. 3-(4,5-dimethylthiazol-2-yl)-2,5-diphenyl-2H-tetrazolium bromide (MTT), dimethyl sulfoxide (DMSO), sodium pyruvate (CH_3_COCOONa), and nicotinamide-adenine-dinucleotide (NADH), 2,4- dinitrophenylhydrazine were supplied by Sigma Aldrich.

### 3.2. Synthesis of Aqueous Suspension of CYS-AgNPs

A 250 mL three-necked round-bottomed flask equipped with a reflux condenser, a dropping funnel, and a gas scrubber were immersed in a water bath (25 °C) placed on a magnetic stirrer. The flask was filled with 160 mL of an aqueous solution of 2.5 mM silver nitrate, purged with argon to eliminate oxygen from the reaction system. Then, 10 mL of deoxygenated sodium borohydride solution (10 mM) was added drop by drop to the silver nitrate solution while stirring vigorously. At this stage of the synthesis, the appearance of a yellow color in the solution indicated the formation of AgNPs. After 2 min following the addition of sodium borohydride, 5 mL of deoxygenated L-cysteine (CYS) aqueous solution (2 mM) was added to the AgNP suspension. Stirring of the reaction mixture under anaerobic conditions was maintained for over 1 h. Then, the ultrafiltration method was applied to purify the suspension from unreacted compounds. The CYS-AgNP suspension was washed using an Amicon 8400 ultrafiltration chamber equipped with a polyethersulfone membrane (Millipore, PBHK07610, Burlington, MA, USA), applying an aqueous solution of nitric acid of pH 4.0. The cleaning procedure was finished when the effluent solution attained the same values of pH and electric conductivity as the applied nitric acid solution [23]. All studies involving the CYS-AgNP suspension were conducted within two weeks.

### 3.3. Physicochemical Characteristic of CYS-AgNPs Dispersed in Suspensions

The mass concentration of CYS-AgNPs was measured by inductively coupled plasma optical emission spectrometry (ICP-OES) using a Perkin-Elmer OPTIMA 2100DV instrument (Wellseley, MA, USA). For this purpose, 400 μL of stock CYS-AgNP suspension was mixed with 1.6 mL of hot nitric acid solution (70%) to dissolve the nanoparticles. The digestion time was one hour according to the protocol developed by Kéri et al. [32]. Afterwards, the mixture was diluted with 48 mL of Milli-Q water (Merck Burlington, MA, USA). The effectiveness of CYS-AgNP dissolution was evaluated using the dynamic light scattering (DLS) technique. The comparison of the results obtained for undissolved suspension and nitric acid-treated solution confirmed that the nanometric structure of CYS-AgNP was destroyed. Additionally, the dissolution of CYS-AgNPs was confirmed in the UV-Vis measurements. The lack of a characteristic band at a wavelength of approximately 400 nm, arising from localized surface plasmon resonance (LSPR), indicated total nanoparticle dissolution (Supplementary Materials, Figure S1).

The morphology, average size, and size distribution of CYS-AgNPs were evaluated using micrographs recorded with a JEOL JSM-7500F electron microscope operating in transmission mode (TEM). The TEM micrographs were analyzed using MultiScan software v.18.03. Histograms were generated by measuring the surface area and diameter of 1000 CYS-AgNPs.

The stability of CYS-AgNPs dispersed in aqueous solutions and culture media was evaluated by measuring their extinction spectra, hydrodynamic diameter (*d*_H_), and rate of silver ion release resulting from the oxidative dissolution process. Extinction spectra of CYS-AgNPs dispersed in solutions with controlled pH, ionic strength, and temperature were recorded using a UV-2600 spectrometer (Shimadzu, Tokyo, Japan). The hydrodynamic diameters of AgNPs were calculated from the Stokes–Einstein relationship based on diffusion coefficient (*D*) measurements carried out using a Zetasizer Nano ZS (Malvern, Worcestershire, UK). The values of hydrodynamic diameters are presented based on the results from the number mode.

The kinetics of silver ion release from CYS-AgNPs dispersed in suspensions with controlled pH, ionic strength, temperature, and concentration of dissolved oxygen (DO) were determined using ICP-OES. The concentration of DO was measured using a COG-1t oxygen probe connected to a CPO-505 oxygen meter (Elmetron, Zabrze, Poland). For this purpose, CYS-AgNP suspensions with controlled properties, maintained at a temperature of 37 °C for a given period of time, were filtered using a regenerated cellulose membrane (Millipore, nominal molecular weight limit 30 kDa) to separate CYS-AgNPs from released silver ions. Collected effluents were fixed before ICP-OES measurements using a nitric acid solution (2% *v*/*v*). It is worth mentioning that three independent samples of effluent were obtained for a given period of time. The concentration of leached silver ions was calculated as the average of three independent experiments.

The electrokinetic properties of CYS-AgNPs, including electrophoretic mobility (*μ*_e_) and zeta potential (*ζ*), were determined based on the electrophoretic light scattering (ELS) technique using a Zetasizer Nano ZS (Malvern). The values of CYS-AgNP zeta potential were calculated using Henry’s model.

### 3.4. Estimation of Interactions between Biotinylated Bovine Serum Albumin (BSA-Bt) with CYS-AgNPs

Biotinylated BSA (BSA-Bt) was prepared according to the protocol described previously [20]. The deposition of BSA-Bt on Nunc Maxi Sorp microtiter plates (Nunc) was evaluated using microplate enzyme-linked ligand sorbent assay (ELISA) [33]. In the next stage of the study, the impact of CYS-AgNPs on BSA-Bt deposition was assessed. For this purpose, BSA-Bt at a concentration of 2.5 nM was deposited on the Nunc plate in the presence of CYS-AgNPs at concentrations ranging from 0.3 to 20 mg L^−1^. The deposition process was conducted for 24 h at a temperature of 4 °C. Afterwards, the wells were washed three times with buffered saline containing 0.05% Triton-X-100 (PBST), and then 200 μL of 0.5% BSA solution in buffered saline was added to each well to block vacant locations on the plate. The incubation process was carried out at 37 °C for 2 h, after which unbound BSA molecules were washed away. In the next stage, streptavidin conjugated with horseradish peroxidase (SA-HRP) was added to each well. The plate with SA-HRP, diluted 4000 times in PBST (50 μL per each well), was incubated at room temperature in the dark for 1 h. Then, the plate was washed five times with PBST. The substrate for HRP was 3,3′,5,5′-tetramethylbenzidine (TMB), of which 50 μL was added to the dried wells and allowed to develop a clear blue color. A quantity of 50 μL of 2 M HCl was added to each well to stop the reaction. Then, 90 μL of each sample was transferred to a clean well, and the absorbance of the solution at a wavelength of 450 nm was measured using an Epoch (BioTek Instruments, Winooski, VT, USA) microplate reader.

### 3.5. Deposition of CYS-AgNPs on Solid Substrate

Electrostatically driven deposition of CYS-AgNPs on oppositely charged surfaces of mica sheets was carried out to obtain CYS-AgNP monolayers of controlled coverage [25]. In the first case, freshly cleaved mica sheets were immersed in the stock CYS-AgNP suspensions, and the ionic strength was increased to 5 × 10^−3^ M using sodium chloride. The diffusion-controlled deposition of CYS-AgNPs on the mica surface was conducted over 12 h to obtain monolayers of the highest coverage. In the second case, mica sheets were modified by poly(diallyldimethylammonium chloride) (PDDA) according to the protocol described previously [25]. Then, the ionic strength and pH of the CYS-AgNPs stock suspension were fixed at 5 × 10^−3^ M and 9.0, respectively, using sodium chloride and Trizma buffer. PDDA-modified mica sheets were placed in CYS-AgNP suspension and stored there for over 12 h. In both cases, the deposition processes were performed in a thermostatted diffusion cell at a temperature of 37 °C.

The CYS-AgNP monolayers formed on bare and PDDA-modified mica sheets were washed in Milli-Q water to remove unbound CYS-AgNPs and the excess electrolytes applied during the deposition. Freshly prepared CYS-AgNP monolayers were characterized using the streaming potential method and scanning electron microscopy (SEM). Additionally, they were applied in further experiments with culture media.

### 3.6. Physicochemical Characteristics of CYS-AgNP Monolayers and Their Interactions with Culture Medium Using Streaming Potential Measurements and Microscopic Imaging

The streaming potential measurements [25,34] were employed to evaluate the electrokinetic properties of formed monolayers, as well as the interactions of immobilized CYS-AgNPs with the proteins present in the culture media used for cell cultivation. In the first step, the CYS-AgNP monolayers were investigated at pH 4.0, 7.4, and 9.0. Subsequently, the monolayers formed under acidic and alkaline conditions were immersed in full medium solutions and stored there for 24 h at a temperature of 37 °C. After this period of time, the monolayers were rinsed with Milli-Q water and investigated using the streaming potential method at pH 7.4. A home-made measuring device described in detail elsewhere [25,32] was utilized in this part of the experiments. The morphology, structure, and coverage of CYS-AgNP monolayers obtained under acidic and alkaline conditions were assessed based on micrographs recorded using a JEOL JSM-750F electron microscope. The monolayers after the streaming potential measurements were attached to holders and then sputtered with a thin layer of carbon to ensure sample conductivity during SEM imaging. The same procedure was applied for the studies of CYS-AgNP monolayers exposed to RPMI 1640 culture medium supplemented with 10% FBS.

### 3.7. Exposure of Lymphocytes to CYS-AgNPs

COLO-720L and HUT-78 cells were cultured in RPMI 1640 culture medium supplemented with 10% fetal bovine serum (FBS) and penicillin–streptomycin at 37 °C in a humidified atmosphere. In typical experiments, 0.2 mL of lymphocytes at a concentration of 1 × 10^6^ cells mL^−1^ was seeded in 96-well plates. Subsequently, the cells were incubated with various concentrations of CYS-AgNPs ranging from 0 to 20 mg L^−1^.

In the first part of the experiments, CYS-AgNPs dispersed in a suspension with a pH of 4.0 were introduced into the full culture medium. In the second stage, the pH of the CYS-AgNP stock suspension was adjusted to 9.0 using Trizma buffer, and this suspension was then introduced into the culture medium. Additional measurements confirmed that the pH of the culture media enriched by CYS-AgNPs, with concentrations up to 50 mg L^−1^ and introduced in the form of both acidic and alkaline suspensions, was equal to 7.4. COLO-720L and HUT-78 cells were also treated with Trizma buffer of the same composition as used in the experiments with CYS-AgNP suspensions at pH 9.0.

The plates with the lymphocytes were then incubated for 24 h at 37 °C. Subsequently, the samples were collected, centrifuged (1000× *g*, 5 min), and analyzed using selected biochemical assays.

### 3.8. Determination of Cell Viability after CYS-AgNP Treatment

The viability of COLO-720L and HUT-78 cells was assessed using 3-(4,5-dimethylthiazol-2-yl)-2,5-diphenyl tetrazolium bromide reduction (MTT) and lactate dehydrogenase (LDH) leakage assays. The MTT assay was employed to evaluate mitochondrial activity in lymphocytes. In brief, 50 μL of MTT solution (5 mg L^−1^) was added to each well containing lymphocytes treated with CYS-AgNPs. The mixtures were then incubated at 37 °C for 2 h, followed by the addition of 0.4 mL of dimethyl sulfoxide (DMSO). After 5 min, the mixtures were centrifuged, and the absorbance of the supernatants was measured at 570 nm using an Epoch microplate reader (BioTek). The absorbance value obtained corresponded to the number of viable lymphocytes. By comparing this value to the absorbance of a sample containing the same number of living cells, the viability of the cells after each treatment was calculated.

The LDH assay was utilized to assess cell membrane damage induced by CYS-AgNPs. For this purpose, supernatants (100 μL) obtained after centrifugation of the lymphocytes were added to a mixture of 0.5 mL of sodium pyruvate (0.75 mM) and 10 μL of NADH (140 μM). These solutions were then incubated at 37 °C for 30 min. Subsequently, 0.5 mL of 2,4-dinitrophenylhydrazine (0.1 M) was added to each mixture. After 1 h, the absorbance of the formed hydrazone was measured at 450 nm using an Epoch microplate reader. The final absorbance value was calculated, taking into account the contribution to the absorbance value from the blank reagent. The LDH leakage from lymphocytes treated with CYS-AgNPs was determined relative to the control, in which LDH release was set to 100% due to membrane disruptions in all cells induced by sonication (15 kHz, 15 min).

### 3.9. Determination of CYS-AgNP Uptake

Inductively coupled plasma optical emission spectrometry (ICP-OES) using Perkin-Elmer OPTIMA 2100DV equipment was utilized to quantify the silver uptake by lymphocytes from CYS-AgNPs. COLO-720L and HUT-78 cells were exposed to CYS-AgNPs at concentrations of 5 and 10 mg L^−1^ for 24 h. Subsequently, sample preparation for ICP-OES measurements followed the protocol developed by Monteiro-Riviere et al. [35] with some modifications. Briefly, after incubation, the lymphocytes were washed with PBS at least three times, trypsinized, and collected by centrifugation. The cell pellets were then treated with 7 mL of 65% HNO3 and mineralized for 15 min at a temperature of 300 °C and a pressure of 45 bar using a Magnum II microwave mineralizer. The samples were diluted using Milli-Q water and the silver content was analyzed using ICP-OES. The concentration of elemental silver in the solution was determined, and the amount of silver taken up by 1 × 10^6^ lymphocytes of both types was calculated. Silver content was also analyzed in the supernatants obtained after centrifugation. Control measurements were conducted for untreated lymphocytes and for the full culture medium. Additionally, similar experiments were performed for lymphocytes exposed to silver ions (delivered in the form of silver nitrate) at concentrations of 5 and10 mg L^−1^. Each sample was analyzed in triplicate and repeated three times independently.

### 3.10. Assessment of Lymphocyte DNA Damage after the CYS-AgNP Exposure

After treating lymphocytes with CYS-AgNPs at a concentration of 10 mg L^−1^, the cell culture containing 1 × 10^6^ cells mL^−1^ was centrifuged. DNA from the cells was extracted using a DNA isolation kit for animal tissues and cell lines (Extractme, EM03-050), Barcelona, Spain). Then, DNA damage was estimated using the standard protocol of the Abcam DNA Damage AP Sites Assay Kit (Abcam, ab65353, Shanghai, China). The relative number of AP sites was estimated by comparing the absorbance value at 450 nm with the absorbance value recorded for the control sample containing untreated lymphocytes at the same cell concentration.

### 3.11. Determination of Lymphocyte Morphology after Exposure to CYS-AgNPs

The lymphocytes, totaling 2 × 10^6^ cells per culture flask, were incubated with CYS-AgNPs at concentrations of 5 and 10 mg L^−1^, CYS-AgNPs were added to the culture medium as suspensions with pH values of 4.0 and 9.0 (adjusted using Trizma buffer). Following 24 h of treatment, COLO-720L and HUT-78 cells were rinsed with PBS buffer. Subsequently, the lymphocytes were detached from the substrate surface using a 1 mM EDTA solution in PBS. The cells were then centrifuged and resuspended in fresh complete culture medium. Next, they were fixed using 2.5% glutaraldehyde in 0.1 M cacodylate buffer (pH 7.4) at 10 °C. After fixation, the cells were post-fixed in 1% osmium tetroxide in cacodylate buffer for 1 h. Following another wash in cacodylate buffer, the lymphocytes were dehydrated in a series of ethanol solutions with concentrations of 50%, 70%, 96%, and 100%. Subsequently, the cells were treated with propylene oxide for 5 min and embedded in Poly/Bed 812. Once the polymerization process was completed, samples were sectioned using a Leica UC7 ultramicrotome. Discs with a thickness of 70 nm were mounted on Formvar-coated single slot copper grids and contrasted with uranyl acetate and lead citrate. Finally, the samples were examined using a JEOL (Tokyo, Japan) 2100HT electron microscope.

### 3.12. Statistical Analysis

The cellular response to CYS-AgNP treatment was investigated in five independent replicates for each biochemical assay conducted. The collected data were averaged to determine the standard deviation. Significant differences compared to the control group were assessed using the SAS analysis of variance (ANOVA) procedure. Statistical analysis of the results obtained from each biochemical assay was performed using the Duncan multirange test, with a significance level set at *p* < 0.05, utilizing PC SAS 8.0 software (SAS Institute, Hong Kong).

## 4. Conclusions

Cysteine-stabilized AgNPs (CYS-AgNPs) were synthesized in the form of aqueous suspension via the reduction of silver ions, delivered in the form of silver nitrate, by sodium borohydride and the functionalization of the AgNP surfaces by cysteine molecules under acidic and anaerobic conditions. The physicochemical characteristics showed that CYS-AgNPs exhibited an isoelectric point at pH 5.1. It was determined that CYS-AgNPs were stable at pH 4.0, 7.4, and 9.0 when the ionic strength did not exceed the value of 10^−2^ M. The stability of CYS-AgNPs dispersed in the full RPMI1640 medium supplemented with 10% FBS was also confirmed by UV-Vis measurements and TEM imaging.

Based on the streaming potential investigations, it was found that the surface of both positively and negatively charged CYS-AgNPs was modified by the adsorption of proteins present in the culture media. Nevertheless, the toxic effect of CYS-AgNPs on COLO-720L and HUT-78 cell lines was significantly stronger when they were introduced to the culture medium as positively charged. CYS-AgNPs(+) reduced cell viability more intensively than negatively charged CYS-AgNPs at the same concentration. Moreover, positively charged CYS-AgNPs strongly damaged the integrity of the cell membrane, which was detected by enhanced LDH release. The genotoxic effect and the morphological changes of the cells were also more intense in the case of treatment with positively charged CYS-AgNPs. The conducted studies revealed that despite CYS-AgNPs being characterized by the same chemical structure of the stabilizing layers, their toxicity towards COLO-720L and HUT-78 cell lines was mainly modulated by their surface charge.

## Figures and Tables

**Table 1:** Selected physicochemical properties of CYS-AgNPs dispersed in aqueous suspensions.

Property/Conditions [Unit] Value	pH		
	4.0	7.4	9.0
plasmon absorption maximum [nm]	401	397	397
nanoparticle size (diameter) [nm] from TEM	18 ± 3	18 ± 2	19 ± 4
polydispersity index (PdI)	0.17	0.11	0.21
diffusion coefficient [10^−7^ cm^2^ s^−1^] *T* = 37 °C, determined from DLS technique at ionic strength: 10^−4^ M NaCl 10^−2^ M NaCl	3.58 ± 0.03 0.52 ± 0.12	3.39 ± 0.03 0.36 ± 0.15	3.79 ± 0.03 0.66 ± 0.13
hydrodynamic diameter [nm] *T* = 37 °C, at ionic strength: 10^−4^ M NaCl 10^−2^ M NaCl	18 ± 3 124 ± 12	19 ± 3 178 ± 12	18 ± 4 18 ± 2
electrophoretic mobility [(μmcm)(Vs)^−1^] *T* = 37 °C, determined from ELS technique at ionic strength: 10^−4^ M NaCl 10^−2^ M NaCl	4.24 ± 0.03 2.11 ± 0.03	−2.45 ± 0.03 −1.19 ± 0.10	−3.11 ± 0.08 −1.55 ± 0.03
zeta potential [mV] *T* = 37 °C, at ionic strength: 10^−4^ M NaCl 10^−2^ M NaCl	69 ± 2 28 ± 4	−39 ± 2 −18 ± 4	−48 ± 3 −23 ± 3

## Data Availability

The data presented in this study are available in the Supplementary Materials.

## Supplementary Materials

The following information can be downloaded at: https://www.mdpi.com/article/10.3390/molecules29153629/s1, Figure S1: UV-Vis spectra. Figure S2; Dependence of (a) diffusion coefficients and (b) hydrodynamic diameters on ionic strength. Figure S3: Dependence of (a) electrophoretic mobility and (b) zeta potential on ionic strength. Figure S4: Kinetics of silver ion release. Figure S5: SEM micrographs of CYS-AgNP monolayers. Figure S6: DNA damage.
