# Supplementary material for: Surface Charge-Modulated Toxicity of Cysteine-Stabilized Silver Nanoparticles
Source: Molecules. 2024 Jul 31;29(15):3629. doi: 10.3390/molecules29153629 (PMC11314351; doi:10.3390/molecules29153629)
Supplement: Supplementary file 1 [file molecules-29-03629-s001.zip › molecules-3085302-supplementary.pdf]

Supporting materials

Surface charge-modulated activity of cysteine-stabilized silver nanoparticles

Magdalena Oćwieja[1*] (magdalena.ocwieja@ikifp.edu.pl), Anna Barbasz[2] (anna.barbasz@up.krakow.pl), Monika Wasilewska[1] (monika.wasilewska@ikifp.edu.pl), Piotr Smoleń[1] (piotr.smolen@ikifp.edu.pl), Dorota Duraczyńska[1] (dorota.duraczynska@ikifp.edu.pl), Bogna D. Napruszewska[1] (bogna.napruszewska@ikifp.edu.pl), Mikołaj Kozak[3] (mikolaj.kozak@doctoral.uj.edu.pl), Adam Węgrzynowicz[4] (adam.wegrzynowicz@pk.edu.pl)

[1]*Jerzy Haber Institute of Catalysis and Surface Chemistry, Polish Academy of Sciences, Niezapominajek 8, PL-30239 Krakow, Poland*

[2] *Department of Biochemistry and Biophysics, Institute of Biology and Earth Sciences, University of the National Education Commission, Podchorazych 2, PL-30-084, Krakow, Poland*

[3] *Jagiellonian University, Faculty of Chemistry, Department of Physical Chemistry and Electrochemistry, Gronostajowa 2, 30-387 Krakow, Poland*

[4] *Faculty of Chemical Engineering and Technology, Cracow University of Technology, 24 Warszawska Street, 31-155 Kraków, Poland*

Oćwieja Magdalena (magdalena.ocwieja@ikifp.edu.pl)
Jerzy Haber Institute of Catalysis and Surface Chemistry, Polish Academy of Sciences
Niezapominajek 8, PL-30239 Krakow, Poland
phone: +48126395112

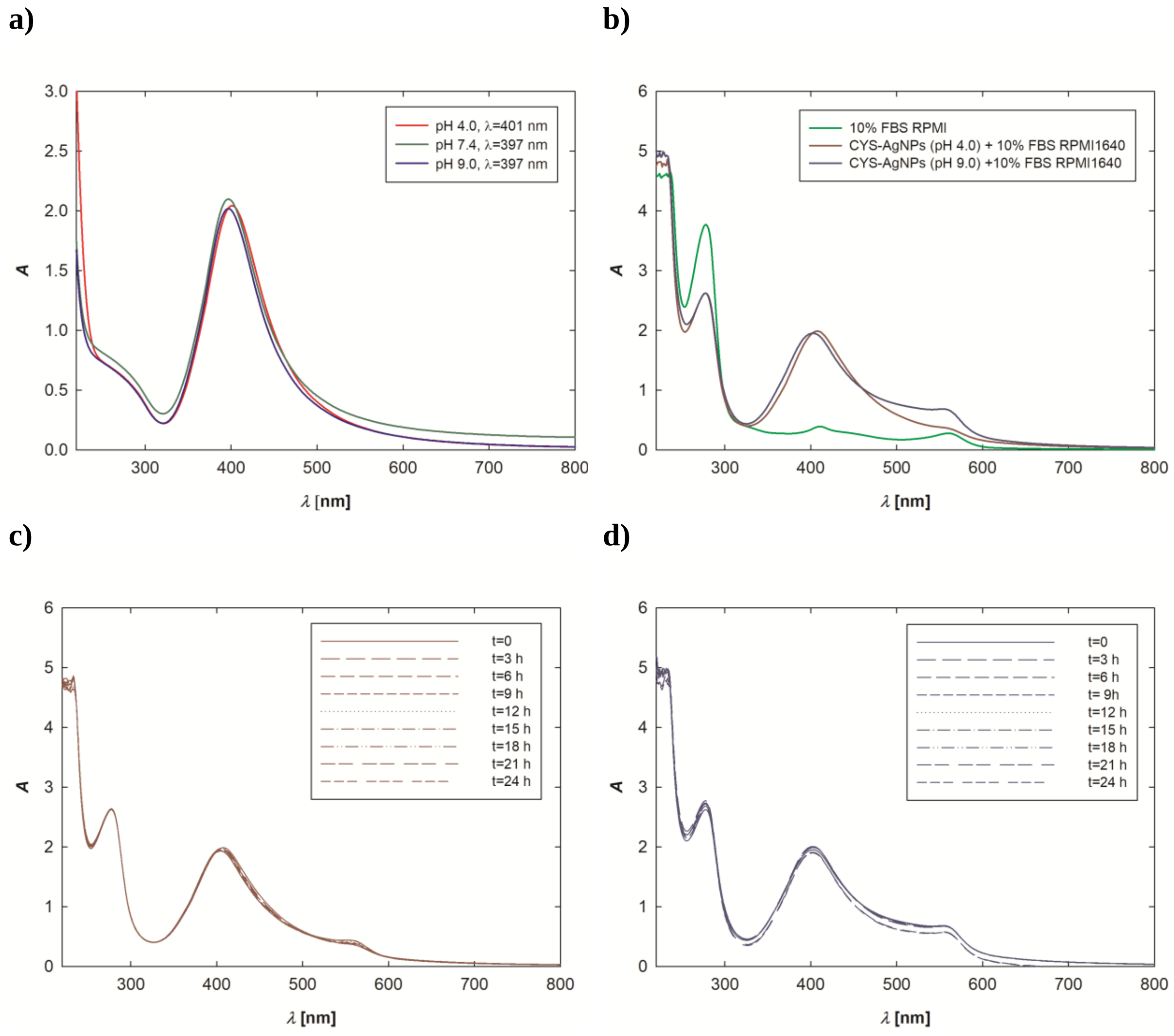

Fig. S1. Extinction spectra of: (**a**) CYS-AgNPs dispersed in aqueous suspensions of diverse pH values, (**b**) CYS-AgNP dispersed in aqueous suspensions of pH 4.0 and 9.0 and then mixed with RPMI1640 medium supplemented with 10% FBS of pH 7.4, (**c**) CYS-AgNPs dispersed in aqueous suspensions of pH 4.0 and then mixed with RPMI1640 medium supplemented with 10% FBS of pH 7.4 recorded after given period of incubation time, (**d**) CYS-AgNPs dispersed in aqueous suspensions of pH 9.0 and then mixed with RPMI1640 medium supplemented with 10% FBS of pH 7.4 recorded after given period of incubation time.

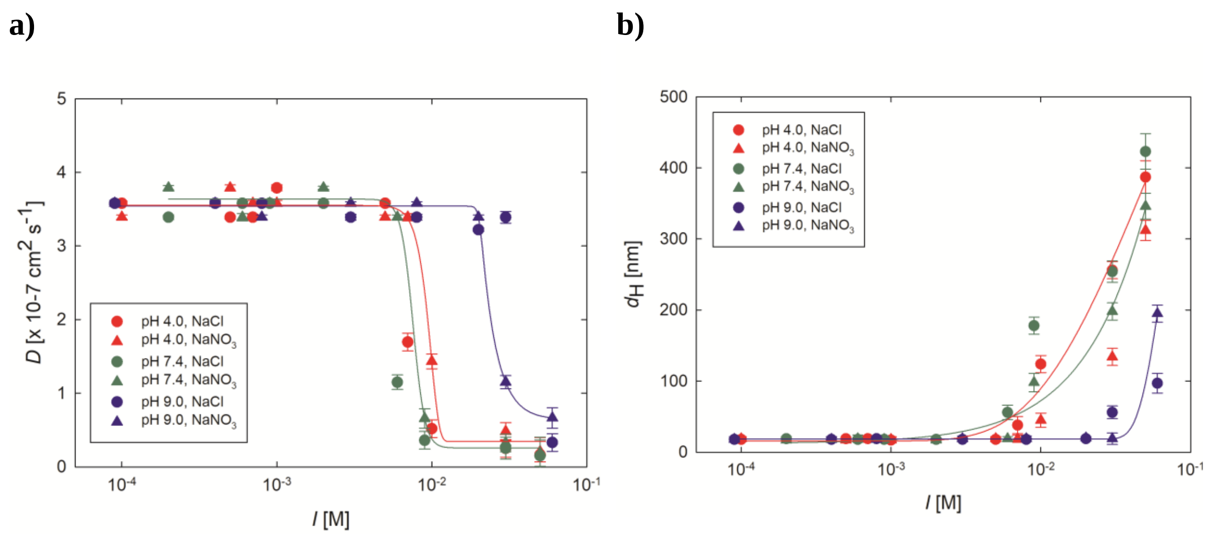

Fig. S2. Dependence of CYS-AgNP (**a**) diffusion coefficient and (**b**) hydrodynamic diameter on ionic strength regulated by the addition of (●,●,●) sodium chloride and sodium nitrate (▲,▲,▲) determined at selected values of pH. The measurements were conducted at *T*=37°C and for CYS-AgNP concentration equal to 50 mg L^{-1}. Solid lines represent nonlinear fits of experimental data.

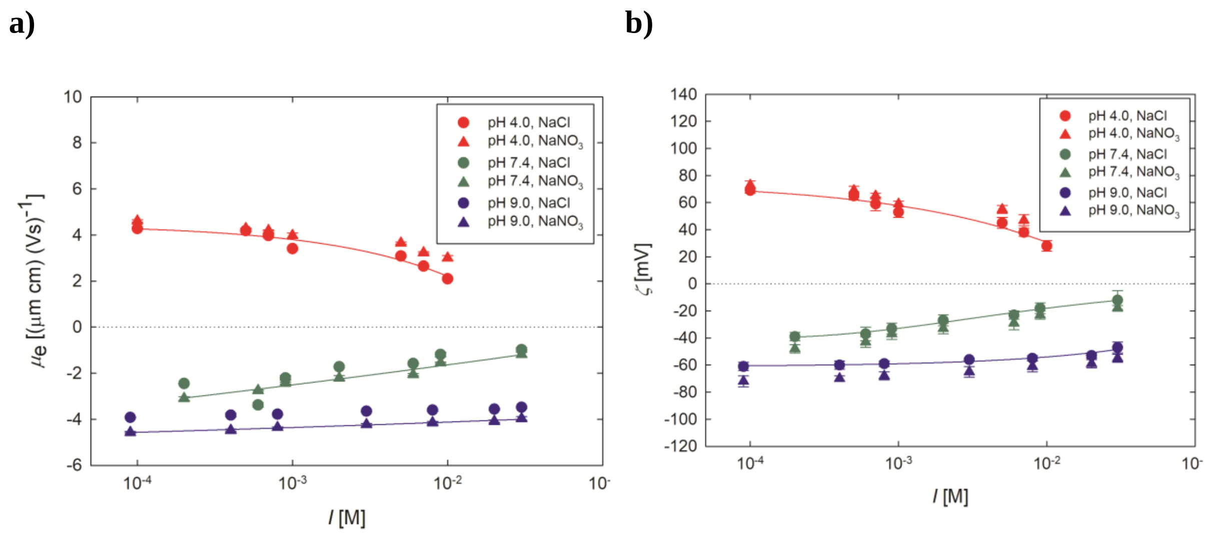

Fig. S3. Dependence of CYS-AgNP **a)** electrophoretic mobility and **b)** zeta potential on ionic strength regulated by the addition of (●,●,●) sodium chloride and sodium nitrate (▲,▲,▲) determined at selected values of pH. The measurements were conducted at *T*=37°C and for CYS-AgNP concentration equal to 50 mg L^{-1}. Solid lines represent nonlinear fits of experimental data.

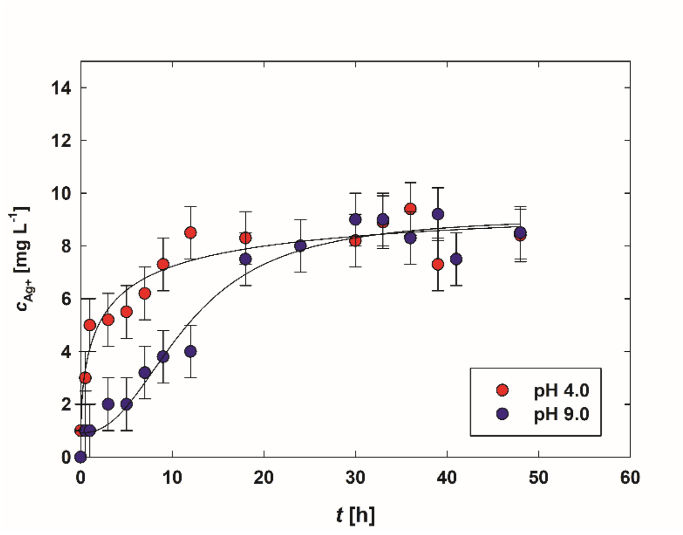

Fig.S4. Kinetics of silver ion release from CYS-AgNPs determined for nanoparticle concentration equal to 50 mg L^{-1}, T=37°C, and DO concentration 5.9 mg L^{-1}, at pH 4.0 and 9.0. Solid lines present nonlinear fits of experimental results.

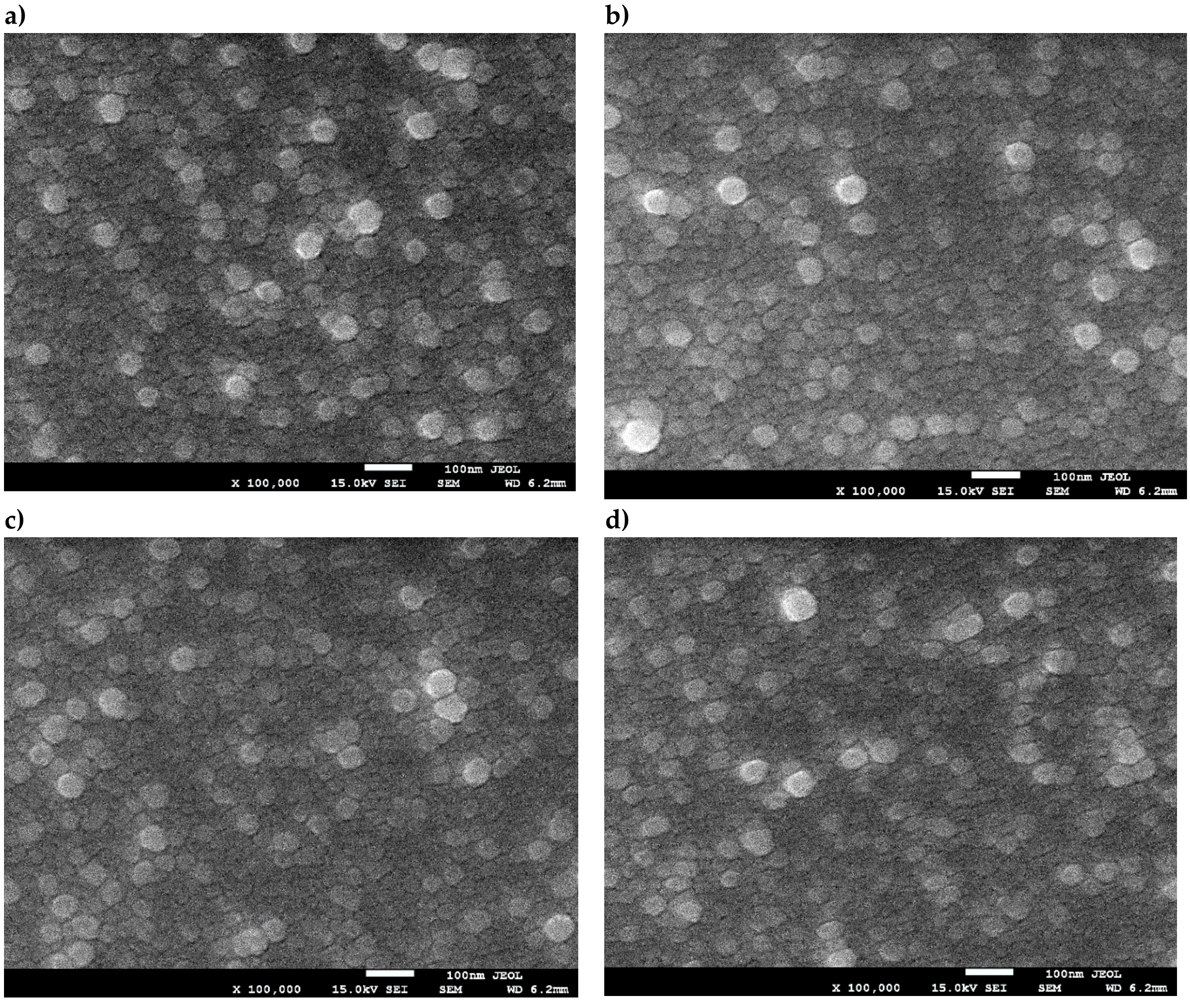

Fig. S5. SEM micrographs of CYS-AgNPs deposited on **(a)** mica using CYS-AgNP suspensions of concentration equal to 50 mg L^{-1}, pH 4.0, ionic strength of $5x10^{-3}$ M NaCl and (deposition time of 12 h, temperature 37°C), (**b)** PDDA-modified mica using CYS-AgNP suspensions of concentration equal to 50 mg L^{-1}, pH 9.0, ionic strength of $5x10^{-3}$ M NaCl and (deposition time of 12 h, temperature 37°C) and SEM micrographs of (**c)** CYS-AgNP monolayers deposited on mica and (**d)** CYS-AgNP monolayers deposited on PDDA-modified mica after 24-h exposure on RPMI1640 medium supplemented with 10% FBS.

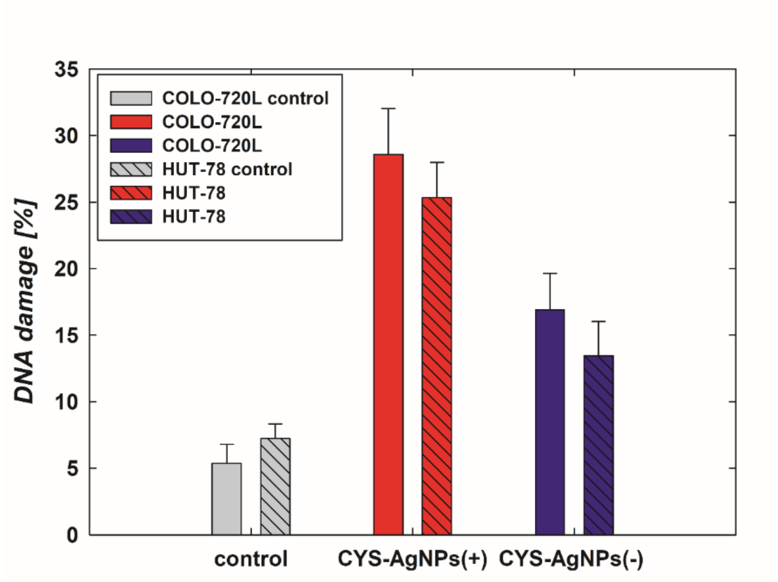

Fig. S6. DNA damage in the cells after 24-hour treatment with 10 mg L^{-1} CYS-AgNPs.
